# Integrated CGH/WES Analyses Advance Understanding of Aggressive Neuroblastoma Evolution: A Case Study

**DOI:** 10.3390/cells10102695

**Published:** 2021-10-09

**Authors:** Diana Corallo, Carlo Zanon, Marcella Pantile, Gian Paolo Tonini, Angelica Zin, Samuela Francescato, Bartolomeo Rossi, Eva Trevisson, Claudia Pinato, Ezequiel Monferrer, Rosa Noguera, Salvador F. Aliño, Maria Jose Herrero, Alessandra Biffi, Elisabetta Viscardi, Sanja Aveic

**Affiliations:** 1 Laboratory of Target Discovery and Biology of Neuroblastoma, Fondazione Istituto di Ricerca Pediatrica Città della Speranza, C.so Stati Uniti 4, 35127 Padova, Italy; d.corallo@irpcds.org (D.C.); m.pantile@irpcds.org (M.P.); gp.tonini@irpcds.org (G.P.T.); 2 Bioinformatics Core Service, Fondazione Istituto di Ricerca Pediatrica Città della Speranza, C.so Stati Uniti 4, 35127 Padova, Italy; c.zanon@irpcds.org; 3 Advanced Diagnostics and Target Discovery in Rare Pediatric Solid Tumors, Fondazione Istituto di Ricerca Pediatrica Città della Speranza, C.so Stati Uniti 4, 35127 Padova, Italy; angelica.zin@unipd.it; 4 Pediatric Hematology, Oncology, and Stem Cell Transplant Center, Department of Woman’s and Child’s Health, University of Padova, Via Gustiniani 3, 35128 Padova, Italy; sam.francescato@gmail.com (S.F.); bart.rossi@gmail.com (B.R.); alessandra.biffi@unipd.it (A.B.); elisabetta.viscardi@unipd.it (E.V.); 5 Clinical Genetics Unit, Department of Woman’s and Child’s Health, University of Padova, Via Gustiniani 3, 35128 Padova, Italy; eva.trevisson@unipd.it (E.T.); claudia.pinato@iov.veneto.it (C.P.); 6 Pathology Department, Medical School, University of Valencia-INCLIVA, 46010 Valencia, Spain; ezequiel.mo.ga@gmail.com (E.M.); Rosa.Noguera@uv.es (R.N.); 7 Pharmacogenetics Unit, Instituto Investigación Sanitaria La Fe and Department Pharmacology, University of Valencia, Avda. Fernando Abril Martorell 106, 46026 Valencia, Spain; alino@uv.es (S.F.A.); Maria.Jose.Herrero@uv.es (M.J.H.); 8 Department of Dental Materials and Biomaterials Research, RWTH Aachen University Hospital, Pauwelsstraße 30, 52074 Aachen, Germany

**Keywords:** Neuroblastoma, recurrent tumor, array CGH, clonal evolution, whole exome sequencing, 3D tumoroids, single nucleotide variants, pharmacogenetics

## Abstract

Neuroblastoma (NB) is the most common extra-cranial malignancy in preschool children. To portray the genetic landscape of an overly aggressive NB leading to a rapid clinical progression of the disease, tumor DNA collected pre- and post-treatment has been analyzed. Array comparative genomic hybridization (aCGH), whole-exome sequencing (WES), and pharmacogenetics approaches, respectively, have identified relevant copy number alterations (CNAs), single nucleotide variants (SNVs), and polymorphisms (SNPs) that were then combined into an integrated analysis. Spontaneously formed 3D tumoroids obtained from the recurrent mass have also been characterized. The results prove the power of combining CNAs, SNVs, and SNPs analyses to assess clonal evolution during the disease progression by evidencing multiple clones at disease onset and dynamic genomic alterations during therapy administration. The proposed molecular and cytogenetic integrated analysis empowers the disease follow-up and the prediction of tumor recurrence.

## 1. Introduction

Neuroblastoma (NB) is an embryonal tumor of the developing sympathetic nervous system [1]. The primary tumors can show a broad spectrum of biological, genetic, and morphological characteristics that make NB one of the most heterogeneous malignancies in children [2]. A search for the genes involved in NB development has revealed a very low number of somatic mutations in primary tumors [3], while the exact cause of NB onset remains unknown. Nevertheless, next generation sequencing has increased the knowledge about NB heterogeneity, improved patients’ risk stratification, and opened new avenues for tailored therapies [4].

Other events such as segmental (e.g., partial chromosome deletions, gains, and amplifications) and numerical (e.g., gain or loss of the entire chromosomes) chromosomal alterations (SCA and NCA, respectively) are closely associated with NB aggressiveness [5]. According to their detected cytogenetic abnormalities, their age at diagnosis, the tumor stage, and their histological characteristics, patients with NB can be stratified into high risk (HR, 50% of all cases), intermediate risk (IR), and low and very low risk (LR) groups [6]. Tumor characteristics strongly correlate with the type of chromosomal aberration, with the prevalence of SCA in HR, and NCA in LR and IR patients [7]. With a prevalence of around 20%, genomic amplification of the MYCN gene (MNA) is one of the most frequent SCAs [8], and the histopathologic evaluation of *MYCN* gene status provides the information required for both patient stratification and treatment protocol assignment [9].

Array comparative genomic hybridization (aCGH) is a gold standard methodology for the analysis of chromosome integrity. Along with fluorescence in situ hybridization, aCGH analysis is a powerful tool for detecting genomic alterations [10]. In NB, allelic losses at chromosomes 1p, 3p, 4p, and 11q, and gains of chromosomes 1q, 2p, and 17q predict poor patient outcomes [11]. Concomitantly, immunohistochemistry (IHC) is a diagnostic tool used to target neural cell adhesion molecule (e.g., NCAM, or CD56) expressing NB cells [12], while CD133 has been associated with cancer stem cell phenotype and chemoresistance in MNA NB [13]. Along with the extracellular matrix (ECM) composition of NB tumors [14], CD133 is proposed as an additional factor that is able to delineate a group of patients with a very poor prognosis. All of these parameters define the clinical portrait of the patient and inevitably influence the incidence of relapses (5–15% in LR/IR cases; ≥50% in HR patients with an overall survival rate of <10%) [15].

In this study, we performed an integrated analysis of aCGH and whole-exome sequencing (WES) data on the genetic material obtained from a rapidly progressing form of NB tumor collected at different time points during the patient’s treatment. Pharmacogenetics was employed to analyze the germline single nucleotide polymorphisms (SNPs) in genes encoding metabolism-related enzymes and drug targets [16] while correlating them with the therapeutic efficacy of the administered drugs. Collectively, genomic, cytogenetic, pharmacogenetics, and biological information have been integrated to assess the molecular rationale for the observed clinical evolution of the analyzed NB and a rapid disease progression.

## 2. Materials and Methods

### 2.1. Patient Information, Tumor Sample Collection, and Primary Cells Maintenance

**Figure 1:**
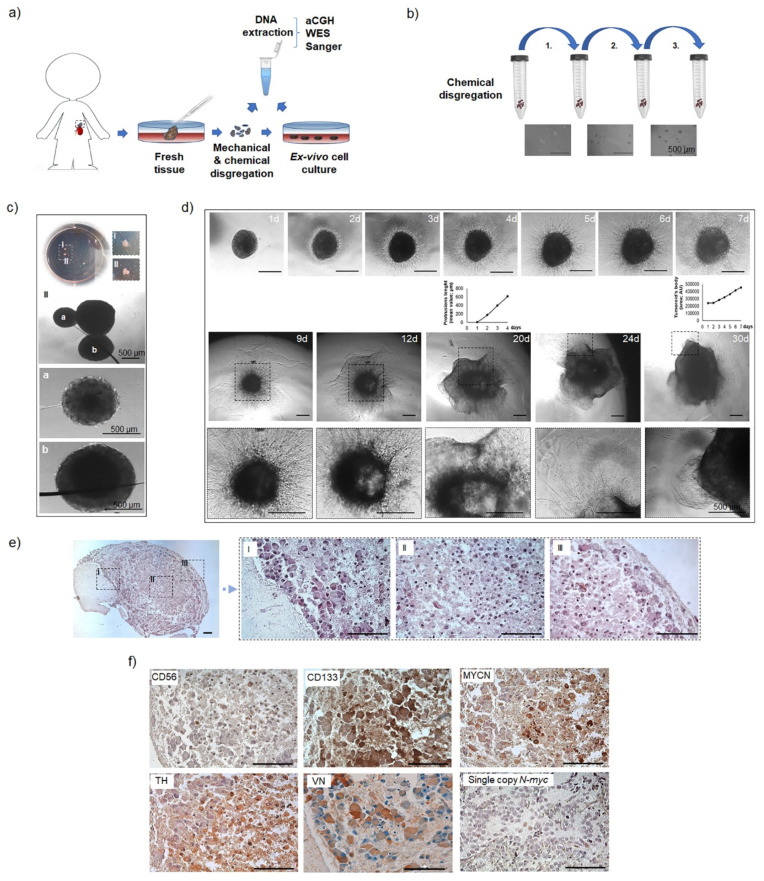
Tumor collection and immunophenotypic characterization. (**a**) Scheme of the tumor tissue processing for molecular biology analyses and ex vivo cell cultivation. (**b**) Three-step procedure of sphere formation assay. Bright field shows sphere formed upon single-cell plating. Scale bar, 500 µm. (**c**) Spontaneously formed spheroids in a petri dish (I and II, top panel) and their single view (white dashed insets). Higher magnifications of the formed 3D structures can be seen in the bottom panels ((**a**) and (**b**)). Scale bars, 500 μm. (**d**) Bright field images of the spontaneously formed spheroids embedded in Matrigel. A time-lapse image was performed until day 30 after embedding. Daily extension of cell invasion frontier from the tumoroid body edge was measured until day 4 (protrusion length is indicated in µm). Growth of the main tumoroid’s body was assessed until day 7 (total area intensity was measured with Fiji and is presented in Arbitrary Units). Scale bar, 500 μm. (**e**) Representative H&E staining of the paraffin-embedded tumoroids. Higher magnifications of different regions of the same tumoroid are indicated (black dashed insets). Scale bar, 200 μm. (**f**) Representative IHC stainings of the paraffin-embedded tumoroids. Sections were stained for CD56 (NCAM), TH (tyrosine hydroxylase), CD133 (prominin-1), Vitronectin (VN) and MYCN (N-myc) proteins. A stage IV, *MYCN* single copy NB primary tumor was used as a negative control for specific MYCN staining. Scale bar, 100 μm.

The informed consent approving the patient-derived material included in the study was obtained from the parents. The case study was conducted following the Declaration of Helsinki, and the protocol was approved by the Ethics Committee of Padua, Italy (Prot. n. 0009761). Malignant tissue samples were obtained by thru cut or after surgical removal and immediately processed according to the type of the study as schematically presented in Figure 1a. Single tumor cells were obtained from recurrent (REC) tumor material after mechanical dissociation and enzymatic digestion (30 min at 37 °C in the digestion medium composed of DMEM/F-12, DNAse (1 mg/mL; Sigma-Aldrich; Milan, Italy)) and collagenase/dispase (1 mg/mL, Roche; Indianapolis, IN, USA), passed through the cell strainer cap (BD Falcon, BD Biosciences; Heidelberg, Germany), and placed in serum-free KnockOut™ DMEM/F-12 medium (Gibco; Milan, Italy) supplemented with 1× B27, 1× N2, bFGF (20 ng/mL, all from Gibco; Milan, Italy), and EGF (20 ng/ml, Cell Guidance Systems; Cambridge, UK) growth factors. A density of 200,000 cells/mL was used for the sphere formation assay to assess the capacity of obtained tumor cells to self-renew after dissociating with TripLE (Gibco; Milan, Italy) [17]. Short-term primary cell cultures allowed for the selection of auto-formed spheroids that were subsequently grown for 30 days without biomimetic ECM support, and 30 days after embedding in Matrigel.

### 2.2. Histological and Immunohistochemical Analyses

The tumoroids were fixed in 4% paraformaldehyde after 60 days of in vitro growth, embedded in paraffin, cut into 7 μm sections, and then subjected to hematoxylin and eosin (H&E) staining according to standard protocols [18]. Immunohistochemistry (IHC) analysis was performed with the following antibodies: mouse monoclonal anti-human CD56 (1:100, SC Biotechnology; Heidelberg, Germany); rabbit polyclonal anti-human CD133 (1:100, Novus Biologicals; Abingdon, UK); rabbit polyclonal anti-human TH (1:100, Cusabio; Houston, TX, USA); rabbit polyclonal anti-human Vitronectin (VT; 1:100, Abcam; Cambridge, UK); rabbit polyclonal anti-human MYCN (1:200, Cusabio; Houston, TX, USA). IHC stains were done manually after an antigen retrieval step (PT Link instrument, Agilent; Santa Clara, CA, USA). All images were acquired with a Zeiss Axio Observer microscope (Oberkochen, Germany).

### 2.3. DNA Extraction, Library Construction, and WES

Genomic DNA (gDNA) was extracted from peripheral blood lymphocytes (PBL) using a QIAmp DNA Mini Kit (Qiagen; Hilden, Germany), while the DNA from the primary tumor (PT), resected residual PT mass (RES), REC tumor, and REC-3D tumoroids were processed using spin filter columns (Invisorb^®^ Spin Tissue Mini Kit, Stratec Molecular GmbH; Berlin, Germany) according to the manufacturers’ protocols. Total gDNA was quantified using a Qubit^®^ dsDNA HS assay (Qubit^®^ 2.0 Fluorometer, Life Technologies; Monza, Italy) and 150 ng were processed by the DNA fragmentation assay (Covaris Model M220, Woburn; MA, USA). The exome library was prepared with the SureSelectXT HS Target Enrichment System (Illumina Paired-End Multiplexed Sequencing Library), subsequently loaded onto an Illumina Next Seq 500/550 High Output Flow Cell Cartridge v2.5 (Illumina; San Diego, CA, USA), and processed with the Illumina Next Seq 500/550. The mean coverage of 100× for PBL and 360× for tumors allowed us to thoroughly explore the variants in malignant tissues. The most frequent *ALK* gene mutations (F1174L and R1275Q), and several randomly selected variants verified by WES, were additionally checked by Sanger sequencing using standard lab protocols [19]. Primers are listed in Supplementary Table S1.

### 2.4. WES Analysis

WES data fastq files were pre-processed with fastp v0.20.0 to remove low-quality stretches of bases at both ends of short reads and then aligned to the reference human genome (hg19) using bwa v0.7.12-r1039 with default options. Allele counts, base calling, and somatic mutation detection were performed using custom scripts based on Samtools v1.3.1. Candidate driver mutations and functional variants were identified using several functional prediction algorithms included in the Annovar software v2018Apr16 and then visually inspected with Samtools ‘tview’.

### 2.5. aCGH Analysis

aCGH was performed on gDNA extracted from PBL, PT, REC, and REC-3D deriving tissue samples. gDNA integrity was additionally assessed on an Agilent 2100 bioanalyzer. aCGH was carried out using the SurePrint G3 Human CGH Microarray 8 × 60 K kit (approximately 60,000 oligonucleotide probes; median probe space 41 kb throughout the genome) (Agilent Technologies; Santa Clara, CA, USA). Control DNA (Promega; Madison, WI, USA) was used as the reference. The arrays were analyzed through an Agilent scanner (G2505C) and Feature Extraction software V.10.1.1.1. A graphical overview of the results was performed using the ‘base’ R statistical software package. DNA sequence information refers to the public UCSC database (Human Genome Browser, February 2009, assembly hg19 (NCBI Build 37.1)).

### 2.6. Pharmacogenetics Study

For the pharmacogenetic study, 1000 µg of DNA was used to genotype all the SNPs included in Supplementary Table S2, in triplicate, using mass spectrometry with MassARRAY device (Agena Bioscience; San Diego, CA, USA) according to the manufacturer’s instructions. The genotyping service was carried out at CEGEN-PRB3-ISCIII. The design of the SNPs included in the panel, the risk assignment for each variant, and the results report are called the VIP Onco study, which is a registered innovation by ©Aliño SF, Herrero MJ Universitat de València/IIS La Fe/HUP La Fe. In brief, the SNPs included in the analysis were chosen according to their relationship with the efficacy and/or toxicity of the drugs most widely employed for pediatric solid tumors, based in PharmGKB (www.pharmgkb.org, accessed on 1 September 2019), drug regulatory agencies (mainly Food and Drug administration, FDA, and European Medicines Agency, EMA), and international pharmacogenetics implementation consortia (mainly Clinical Pharmacogenetics Implementation Consortium, CPIC, and Dutch Pharmacogenomics Working group, DPWG). Only those SNP–drug pairs with the highest levels of evidence were selected to be included in the panel.

### 2.7. Statistical and Clonal Analysis

The statistical analyses and visualizations were performed with R statistical software v3.5.2: packages ‘base’ and ‘stats’ for the common statistical tests; ‘prcomp’ for Principal Component Analysis (PCA); ‘dbscan’ for cluster identification; ‘graphics’, ‘riverplot’ and ‘fishplot’ for clonal composition and evolution modeling; and visualization, ‘euler’, for Venn-Euler plot. Bedtools v.2.25.0 was used for mapping features on genomic intervals.

## 3. Results

### 3.1. Case Description

A two-year-old child was hospitalized with symptoms of fever, stomach pain, and constipation. An abdominal mass of a hard consistency was revealed by palpation and magnetic resonance, and irregular margins were present at the left hemi-abdomen with extension up to 2–3 cm below the transverse umbilical line (Supplementary Figure S1a). The presence of a retroperitoneal primary mass (12 × 11 × 8.5 cm) containing inhomogeneous fat and calcifications zones resulted in the dislocation of the left kidney and aorta toward the right side. aCGH was performed on DNA extracted from the PT typifying a gain of 17q, a loss of 11q, while an MNA was not found (Supplementary Figure S1b). The former chromosomal alterations are recognized as a marker of poor prognosis in NB [20]. Histologically, the tumor was poorly differentiated with intense mitotic karyorrhexis index positivity. No metaiodobenzylguanidine uptake was reported and no sign of disseminated NB cells was observed in the bone marrow (BM) biopsy or BM aspirate (data not shown). An evaluation of other clinical parameters gave the following values: HVA/Cr (417 mmol/mol), VMA/Cr (208 mmol/L), elevated ferritin and NSE levels, and hypertension (180/90 mmHg). Based on the clinical and histological evaluation, the tumor was classified as L2 stage.

During induction chemotherapy, the patient was treated with a rapid COJEC according to the Society For Pediatric Oncology European Neuroblastoma HR-NBL-1 protocol [21] that led to substantial tumor mass reduction and overall improvement of the clinical parameters and general patient’s condition. Subsequently, RES mass (post-COJEC) was collected for the analysis after surgical intervention, while the patient’s clinical and biological data advised the introduction of the European Low and Intermediate Risk Neuroblastoma Protocol (LINES; NCT01728155). After an excellent initial response to the administered therapy, rapid disease progression occurred leading to uncontrolled tumor growth and the patient’s demise. Considering the peculiarity of the clinical course of the disease, we investigated the genetic background that sustained poor response to adopted treatment and disease recurrence.

#### 3.1.1. The 3D In Vitro Study

To explore whether the aggressive malignant cell behavior was maintained in vitro, the tumor material obtained from the REC mass (post-LINES) was used for the cell expansion ex vivo (Figure 1a). The sphere formation assay sustained the intrinsic self-renewal property of the 3D structures and implied for cancer stem cells’ presence (Figure 1b). After three weeks of culture, pre-dissociated single cells maintained the capability to form spheroids spontaneously (Figure 1c), and during the following 30 days established compact 3D structures. To explore their invasive capacities, we embedded them in the biomimetic ECM (Figure 1d). Two days after the embedding, a clear invasive cell front developed around the spheroid body. Radially organized cell extrusions were formed progressively occupying the entire volume of free ECM and served as a leading trail for a single cell migration, corroborating their pro-invasive features (Supplementary Figure S1c). Such behavior led to the in vitro tumor outgrowth and the generation of thick tumoroid-like structures (named REC-3D) (Figure 1d). Histological evaluations of the tumor mass collected after 60 days of in vitro growth confirmed that the REC-3D tumoroids displayed features of poorly differentiated/differentiating neuroblasts (Figure 1e) with positive immunoreactivity for CD56, tyrosine hydroxylase (TH), and particularly for the CD133 stem cell marker (Figure 1f).

Moreover, a high rate of accumulated intracellular VN abundantly expressed in the ECM of aggressive NB [22] was present, highlighting the pro-invasive capabilities of the tumor cells. Notably, positive immunoreactivity toward MYCN proteins was detected in the REC-3D tumoroids, implying its triggered MYCN overexpression during the therapy course. Intriguingly, accelerated in vitro growth of tumor cells anticipated the clinical manifestation of the disease progression.

#### 3.1.2. Genomic Analyses

**Figure 2:**
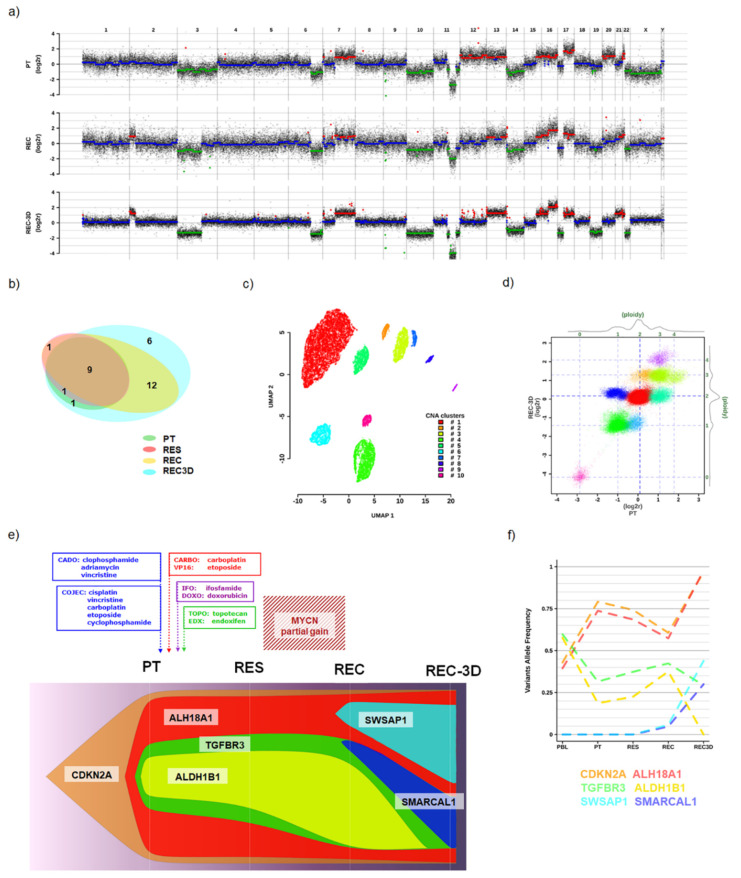
Analysis of tumor specific genetic alterations during disease progression. (**a**) Whole-genome array comparative genomic hybridization (aCGH) profiles of the primary tumor biopsy (PT), recurrent disease (REC), and 3D tumoroids deriving from REC material (REC-3D). Chromosome names on top and log2 of tumor/normal signal intensities ratio on the Y-axis. Log2r values averaged across 50 kb overlapping windows are represented by black dots, while their segmented values represent gains (red), losses (green), and no change (blue). (**b**) Euler diagram indicates the number of somatic mutations identified in each sample (PT, RES, REC, and REC-3D). (**c**) UMAP clustering of segmented log2r values analyzed across PT, REC, and REC-3D samples. (**d**) Dot plot of the segmented log2r aCGH values in the PT vs. REC-3D samples (log2r scales at the bottom and left, ploidy scales at the top and right); each segmented value is color-coded according to the CNAs-derived UMAP clustering of panel b. (**e**) Fish plot of clonal analysis based on germinal and somatic SNVs (WES) stratified by the CNAs-derived (aCGH) UMAP clusters. The treatment scheme is emphasized on top along with the temporal window harboring the *MYCN* gain. Prominent genes were singled out to identify each evolving clone. (**f**) Allele frequency changes of variants characterizing each evolving clone across the analyzed samples.

The aCGH analysis showed that the genome of all the tested specimens shared some common events along with acquiring new alterations during the disease progression. Circulating tumor DNA of approximately 166bp length (data not shown) was not traced in PBL which showed a euploid profile (Supplementary Figure S1b). Diverse CNAs were detected in the DNA obtained from PT, REC, and REC-3D samples suggesting for the genome instability. Numerous chromosomes (3, 6, 7, 10, 11, 12, 13, 14, 15, 16, 17, 20, and 22) were found altered in the PT and during the disease progression (such as losses: 3p, 6q, 11q, 22q, and gains: 7q, 15q, 17q) (Figure 2a). Among these alterations, a loss in 11q is particularly remarkable since it generated a nullisomy of *ATM*, a gene involved in DNA repair. The REC and REC-3D acquired a partial gain in 2p, affecting the regions including *MYCN*, and two atypical SCAs (loss of 3p and gain of 16q). The gain in 2p was confirmed by quantitative PCR (qPCR) analysis (Supplementary Table S1), thus explaining the observed MYCN protein overexpression (Figure 1f). Genome-wide CNA profiles showed a high correlation between REC and REC-3D, with both sharing key attributes with PT (Pearson correlation coefficient: 0.95; r^2^: 0.90), thus indicating that the tumoroid structures maintained the essential copy number features of the parental tumor but attained additional rearrangements such as 17p and 19 losses.

To pursue the possibility of an existent predisposition toward the observed modest efficacy of intensive chemotherapy and radiation, we performed a pharmacogenetics investigation detecting risk alleles in 23/61 SNPs related to drugs currently used in pediatric oncology (for a complete list of the SNPs, see Supplementary Table S2). Several SNPs in the PBL were potentially relevant for the final response to the COJEC and LINES protocols. The same SNPs were confirmed by WES analysis (bold capital letters; Table 1), thus reinforcing the possibility to evaluate the pharmacogenetics risk alleles in tumor-derived material.

An extended analysis on the RES and REC tumor material showed additional variations worsening the SNPs risk pattern. In addition, WES pointed out the risk alleles with frequencies of 5–10% possibly involved in defining the type of response to therapy (bold lowercase letters; Table 1). These findings imply a probable cumulative burden of the SNPs (Supplementary Table S3) that could lead to a limited response to therapy.

To assess the mutational profile of the malignant tissue, WES analysis was performed on DNA from PBL, PT, RES, REC, and REC-3D. A total of 30 somatic variants were identified, most of which were shared among tumor samples (Figure 2b): 11 in PT, 11 in RES, 22 in REC, and 29 in REC-3D (Tables S4 and S5). Twenty-two mutations were exonic (17 non-synonymous, five synonymous), seven intronic/intergenic, and one was confirmed in the 5′UTR. The most frequent *ALK* mutations (F1174L and R1275Q) were not found in any of the analyzed specimens (data not shown). Several heterozygous germline variants that showed frequency shifts among samples were validated by Sanger sequencing (Supplementary Table S1).

By applying the Uniform Manifold Approximation and Projection approach (UMAP) to CNAs identified by aCGH across PBL, PT, REC, and REC-3D (WES-derived segments are highly correlated with aCGH data, thus giving remarkably similar outcomes), we identified clusters of distinctive genomic segments (Figure 2c) with coordinated ploidy switches in recurrent samples (Figure 2d and Supplementary Figure S1d). The coordinated allele frequencies’ shifts in germline SNPs suggest their connection to CNAs fluctuations, envisaging a scenario with a selection of deleterious alleles upon numerical imbalances including those that are copy-neutral. This prompted us to attempt an integrated CNA–SNV modeling of the tumor clonal composition and dynamics considering the interplay between all the identified genomic alterations. By combining the inferred CNAs- and SNVs-derived clonal models, we obtained a synthesis of the possible mass composition and clonal evolution (Figure 2e and Supplementary Figure S1e) while taking into consideration the allele frequency changes of variants characterizing each evolving clone (Figure 2f). The synthetic model foresees the presence of six key clones, hereafter named after single genes harboring specific variants, including four nested ones originating in PT, three persisting in all tumor samples and characterized by variants in *CDKN2A*, *ALDH18A1*, and *TGFBR3*, respectively, while the fourth with a change in *ALDH1B1* was not detected in REC-3D (Figure 2e). Then, two independent clones featuring somatic variants arose in the REC sample and propagated to REC-3D: one identified by a mutation in *SMARCAL1* and nested in the *TGFBR3* background, while the second harbored within the *ALDH18A1* clone with mutations in the *SWSAP1* gene (Figure 2e,f). Notably, the nonsynonymous rare germline alleles (minor allele frequencies ranging between 0.01% and 5%) in *ALDH18A1* [23], *CDKN2A* [24], *TGFBR3* [25], and *ALDH1B1* genes had predicted unfavorable effects on protein function and were previously associated with NB, indicating a possible role for an underlying oligogenic mechanism [26]. Altogether, the combination of aCGH and WES data allowed the shortlisting of the putative causal variants required for clone selection within the tumor.

## 4. Discussion

The therapeutic approaches currently used in the treatment of HR patients with NB unfortunately show limited effectiveness stemming from an insufficient understanding of the biology of the disease [27]. The huge heterogeneity among NB tumors demands a more accurate and detailed diagnosis able to identify smaller, more clinically homogeneous groups of patients or individuals so to tailor more specific and more effective therapeutic regimens.

The possibility of analyzing more specimens from the same patient during the disease course allows for a more complete follow-up of the clinical progression [28]. Advances made in the performing standards, and economically sustainable diagnostic analyses such as aCGH, allow for the detection of small chromosomal imbalances reaching a greater resolution when compared to cytogenetic analyses [29]. In our research, the analysis of aCGH data alone or in combination with the WES recognized a driver in chromosomal alterations spanning the time window between the diagnosis and disease recurrence. By considering the intermediate time points of the disease progression, and the 3D tumoroid structures spontaneously formed from a recurrent tumor mass, we were able to explore the NB clones and define the related dynamics of their evolution during the treatment. By unveiling the combinations of copy number imbalances in the PT, REC, and REC-3D ex vivo tumor material, while correlating them with germinal SNPs and somatic variants, we achieved new insights into the nature of developing tumor masses. Some of the reported genomic alterations were atypical (a loss of 3pq and gain of 16q), while others were already reported for their hazardous potentials such as the gain of 2p and the loss of 11q and 6q [30]. The loss of chromosome 11q harboring the *ATM* gene is frequently seen in tumors without MNA and is an unfavorable prognostic marker for the patients [20]. The recognition of novel alterations as strong clinical risk group classifiers is the key step toward developing new modes of targeted therapy. In the analyzed case, aCGH was instrumental in revealing the dynamics of the disease progression during therapy.

Based on the aCGH/WES data, we discovered that most of the recurrent clones emerged early during the disease progression and persisted throughout the therapy treatment while being flanked by new somatic variants. The synchronous involvement of CNAs and germinal SNPs frequency fluctuations, including those which were copy-neutral and exceedingly rare, suggests an initial generation of clonal diversity that is mainly driven by imbalances affecting disadvantageous alleles. Such mechanisms could also explain part of the observed multidrug resistance, as the pharmacogenetics results strongly suggest. Additional newly arising somatic SNVs at relapse could have helped in determining the outcome. The most frequent mutations in the *ALK* gene were not found, excluding the genetic burden toward this tyrosine kinase. Pharmacogenetics analyses provided an advanced snapshot of the patient’s response to chemotherapy, suggesting the strong predictive potential of this method in clinical routine. The pharmacogenetics approach also revealed a particular result of interest: the patients bore the AA genotype at SNP rs1801133 in the *MTHFR* gene in the PBL sample but also in all the other specimens analyzed by WES. This variant has been previously linked with MNA [31], and although the actual causal link is not yet clear, it is remarkable that while this patient originally had a non-MNA primary tumor, after the chemotherapy resistance a gain of the 2p locus with *MYCN* gene also occurred.

The present study allowed us to better comprehend the genomic abnormalities that occurred during the disease progression. With more articulated aCGH analysis, we were also able to determine the clonal heterogeneity and to follow the ongoing evolution of the candidate genetic lesions during the patient’s cure. The high rate of chromosomal instability and the acquired mutations found in the recurrent mass correlated with an unfavorable clinical picture, with the observed rapid tumor progression, with the aggressiveness, and with the drug-resistant phenotypes [5]. In fact, the results of our study sustain the possibility of achieving an improved prognosis by more systematic genome analysis. Moreover, they affirm that we might be able to anticipate the disease progression by studying in vitro culture behavior and by longitudinally analyzing the DNA imbalances. The most intriguing feature of the 3D tumoroids was the observed burst of the in vitro growth that anticipated a dramatic escalation of the disease clinical presentation.

## 5. Conclusions

Collectively, we have showed that besides providing information on CNAs, aCGH may be instrumental, upon the integration with mutational information, in refining the clonal dynamics that allow for an improved disease progression monitoring.

## Figures and Tables

**Table 1:** SNPs correlated with drug resistance. The asterisk highlights drugs included in the treatment protocols adopted for the patient. Bold capital letters indicate the presence of the risk SNPs alleles found in the analyzed samples (a lowercase letter indicates the additional change of the allele observed by WES with the frequency <5%). Note: The genetic variants included in this study represent SNPs. The chosen SNPs and the recommendations provided are based on the highest scientific level of evidence (1 and 2) according to Pharmacogenetics Knowledge Base (PharmGKB), drug regulatory agencies (FDA, EMA), and international pharmacogenetics consortia (mainly CPIC and DPWG). (www.pharmgkb.org, accessed on 1 September 2019).

			Genotypes		SNP Array	WES				
Drug	Gene	SNP	NO Risk	Risk	PBL	PBL	PT	RES	REC	REC-3D
Azathioprine	TPMT	rs1800462	CC	**CG,GG**	CC	CC	CC	CC	CC	CC
	TPMT	rs1800584	CC	**CT,TT**	CC	CC	CC	CC	CC	CC
	TPMT	rs1142345	TT	**TC,CC**	TT	TT	TT	TT	TT	TT
	TPMT	rs1800460	CC	**CT,TT**	CC	CC	CC	CC	CC	CC
	NUDT15	rs116855232	CC	**CT,TT**	CC	CC	CC	CC	CC	CC
Carboplatin *	ERCC1	rs11615	GG	**AG,AA**	**AA**	**AA**	**AA**	**AA**	**AA**	**AA**
	ERCC1	rs3212986	AA	**AC,CC**	**CC**					
	GSTP1	rs1695	GG	**AG,AA**	**AA**	**AA**	**AA**	**AA**	**AA**	**AA**
	MTHFR	rs1801133	AA	**AG,GG**	AA	AA	AA	AA	AA	AA
	NQO1	rs1800566	GG	**AG,AA**	**AG**					
	XRCC1	rs25487	CC	**CT,TT**	CC			CC		
Cyclophosphamide *	GSTP1	rs1695	AA,AG	**GG**	AA	AA	AA	AA	AA	AA
	SOD2	rs4880	AA	**AG,GG**	**GG**		**GG**	**GG**	**a-GG**	**GG**
	TP53	rs1042522	CC	**CG,GG**	CC	CC-**g**	CC-**g**	CC-**g**	CC-**g**	CC-**g**
Cisplatin *	ERCC1	rs11615	GG	**AG,AA**	**AA**	**AA**	**AA**	**AA**	**AA**	**AA**
	ERCC1	rs3212986	AA	**AC,CC**	**CC**					
	GSTP1	rs1695	GG	**AG,AA**	**AA**	**AA**	**AA**	**AA**	**AA**	**AA**
	MTHFR	rs1801133	AA	**AG,GG**	AA	AA	AA	AA	AA	AA
	NQO1	rs1800566	GG	**AG,GG**	**AG**					
	TP53	rs1042522	CC	**CG,GG**	CC	CC-**g**	CC-**g**	CC-**g**	CC-**g**	CC-**g**
	TPMT	rs1800462	CC	**CG,GG**	CC	CC	CC	CC	CC	CC
	TPMT	rs1800584	CC	**CT,TT**	CC	CC	CC	CC	CC	CC
	TPMT	rs1142345	TT	**TC,CC**	TT	TT	TT	TT	TT	TT
	TPMT	rs1800460	CC	**CT,TT**	CC	CC	CC	CC	CC	CC
	XRCC1	rs25487	CC	**CT,TT**	CC					
	XPC	rs2228001	TT	**GT,GG**	TT					
Doxorubicin *	NQO1	rs1800566	GG	**AG,GG**	**AG**					
Etoposide *	DYNC2H1	rs716274	AA	**AG,GG**	**AG**					
Opioids	ABCB1	rs1045642	AA,AG	**GG**	AG	AG	**g-**AG	**g-**AG	AG	**g-**AG
Irinotecan	C8orf34	rs1517114	GG	**CG,CC**	GG					
	SEMA3C	rs7779029	TT	**CT,CC**	TT					
	UGT1A1	rs4148323	GG	**GA,AA**	GG	GG	GG	GG	GG	GG
Mercaptopurine	TPMT	rs1800462	CC	**CG,GG**	CC	CC	CC	CC	CC	CC
	TPMT	rs1800584	CC	**CT,TT**	CC	CC	CC	CC	CC	CC
	TPMT	rs1142345	TT	**TC,CC**	TT	TT	TT	TT	TT	TT
	TPMT	rs1800460	CC	**CT,TT**	CC	CC	CC	CC	CC	CC
	NUDT15	rs116855232	CC	**CT,TT**	CC	CC	CC	CC	CC	CC
Methotrexate	ABCB1	rs1045642	GG	**AG,AA**	**AG**	**AG**	**g-AG**	**g-AG**	**AG**	**g-AG**
	SLCO1B1	rs11045879	CC	**CT,TT**	**CT**					
	MTHFR	rs1801133	GG	**AA,AG**	**AA**		**AA**	**AA**	**AA**	**AA**
	MTRR	rs1801394	AA	**AG,GG**	**GG**	**GG**	**a-GG**	**GG**	**GG**	**a-GG**
	ATIC	rs4673993	CC,CT	**TT**	CT					
Ondansetron	ABCB1	rs1045642	AA	**AG,GG**	**AG**	**AG**	**g-AG**	**g-AG**	**AG**	**g-AG**
Thioguanine	TPMT	rs1800462	CC	**CG,GG**	CC	CC	CC	CC	CC	CC
	TPMT	rs1800584	CC	**CT,TT**	CC	CC	CC	CC	CC	CC
	TPMT	rs1142345	TT	**TC,CC**	TT	TT	TT	TT	TT	TT
	TPMT	rs1800460	CC	**CT,TT**	CC	CC	CC	CC	CC	CC
	NUDT15	rs116855232	CC	**CT,TT**	CC	CC	CC	CC	CC	CC
Vincristine *	CEP72	rs924607	CC,CT	**TT**	CC					

*—drugs included in the protocols used for the patient’s cure.

## Data Availability

The datasets generated during the study are available from the corresponding author upon request.

## Supplementary Materials

The following are available online at https://www.mdpi.com/article/10.3390/cells10102695/s1. Supplementary figure legends. Figure S1. Data summary. (a) Computed tomography (CT) images at the moment of diagnosis. Yellow circles indicate localized tumor mass on the dorsal and ventral sections of the CT scans. (b) aCGH profile of the PT at the moment of diagnosis and the corresponding PBL. (c) Invasive cell front developed around the spheroid body. Radially organized fibers that serve as a leading trail for a single cell migration are marked with white arrows. Scale bar, 100 µm. (d) Dot plot of the segmented log2r aCGH values in the REC vs. REC-3D samples (log2r scales at the bottom and left, ploidy scales at the top and right). Each segmented value is color-coded according to the CNAs derived UMAP clustering of Figure 2b. (e) aCGH whole-genome profiles of the PT, REC, and REC-3D tumor material. Chromosome names on top and log2 of tumor/normal signal intensities ratio on the Y-axis. Log2r values averaged across 50 kb overlapping windows are represented by black dots, while their segmented values represent CNAs derived UMAP clusters with the same color code used in Figure 2b. Supplementary Table Legends. Table S1. List of primers used in this paper for real-time qPCR and Sanger sequencing. Table S2. Pharmacogenetics analysis. Summary of the SNPs currently correlated with response to the most frequently used antineoplastic drugs (according to PharmGKB 1 and 2 levels of evidence in Clinical Annotations; Drug Labels; and Guidelines from Pharmacogenetics International Experts Consortia). Table S3. Cumulative effects of SNPs in tumor specimens. Protocols used to treat the patient are listed. Final genotype numbers are a sum of all risk genotypes found in the samples analyzed by WES. Brackets indicate that risk genotype was found in <5% of risk-related alleles. Table S4. WES data. Excel spreadsheet with summary information on somatic mutations identified by WES. Table S5. WES data. Excel spreadsheet with detailed information on germline variants identified by WES.
