# Supplementary material for: Integrated CGH/WES Analyses Advance Understanding of Aggressive Neuroblastoma Evolution: A Case Study
Source: Cells. 2021 Oct 9;10(10):2695. doi: 10.3390/cells10102695 (PMC8534916; doi:10.3390/cells10102695)
Supplement: Supplementary file 1 [file cells-10-02695-s001.zip › Suppl Table S1.pdf]

Primer name	Primer sequence (hg19)	Analysis
**ATM_ Forward**	5'- ACGGAAGTTGCATTGTGTCA -3'	qPCR
**ATM_ Reverse**	5'- ATGGCTCCAAGTAAGCCAAA -3'	qPCR
**GAPDH_ Forward**	5'- CGACCACTTTGTCAAGCTCA -3'	qPCR
**GAPDH_ Reverse**	5'- CCCTGTTGCTGTAGCCAAAT -3'	qPCR
**ABL1_ Forward**	5'- ATACGAAGGGAGGGTGTACCA -3'	qPCR
**ABL1_ Reverse**	5'- TTATCAATCCGACAGCAGGAC -3'	qPCR
**ALK_ Forward**	5'- ATGACCTTGTGGCTTTCAGG -3'	qPCR
**ALK_ Reverse**	5'- GACTGACCCTCCCTCCTTGT -3'	qPCR
**MYCN_ Forward**	5'- CTTCGGTCCAGCTTTCTCAC -3'	qPCR
**MYCN_ Reverse**	5'- TCTTGCCTGCAATTTTTCCT -3'	qPCR
**RET_ Forward**	5'- AGATCCACTGTGCGACGAG -3'	Sanger seq
**RET_ Reverse**	5'- AAATGGGGGCAGAACACAG -3'	Sanger seq
**MTHFR_ Forward**	5'- TGGGGTGGAGGGAGCTTAT -3'	Sanger seq
**MTHFR_ Reverse**	5'- TCCCTGTGGTCTCTTCATCC -3'	Sanger seq
**ERBB2_ Forward**	5’- CCCCTAATGGGTCACCTTCT -3'	Sanger seq
**ERBB2_ Reverse**	5'- GGTCCTGGTCCCAGTAATAGAG -3'	Sanger seq
**ADAMTSL2_ Forward**	5'- CGAGAGCTTCTTCGTGGATT -3'	Sanger seq
**ADAMTSL2_ Reverse**	5'- AGGCCTCTGACTTTGTGCTC -3'	Sanger seq
**MMP25_ Forward**	5'- CCCTGACATGGAGGTAGGT -3'	Sanger seq
**MMP25_ Reverse**	5'- GGTAGCTGTCCTGGTGGAA -3'	Sanger seq
**NLRP1_ Forward**	5'- CCTGACGTTTCATCCAGAGG -3'	Sanger seq
**NLRP1_ Reverse**	5'- GCCCCTCTACTTCAACATGG -3'	Sanger seq
**ALK (ex23)_Forward**	5'- GCAAGATTCTGGGTTTAGGC -3'	Sanger seq
**ALK (ex23)_Reverse**	5'- CCATCGAGGAACTTGCTACC -3'	Sanger seq
**ALK (ex25)_Forward**	5'- GGCTGTTTCTCTCACACTGAAG -3'	Sanger seq
**ALK (ex25)_Reverse**	5'- CTTCGGGCATGGTCACTAAT -3'	Sanger seq
