# Supplementary material for: Integrated CGH/WES Analyses Advance Understanding of Aggressive Neuroblastoma Evolution: A Case Study
Source: Cells. 2021 Oct 9;10(10):2695. doi: 10.3390/cells10102695 (PMC8534916; doi:10.3390/cells10102695)
Supplement: Supplementary file 1 [file cells-10-02695-s001.zip › Suppl Table S3.pdf]

Supplementary Table S4. Sum of the pharmacogenetic risk_genotypes detected in different specimens.

	risk_genotypes / total_genotypes			risk_genotypes / total_genotypes				
	Protocol	WES	Drug	WES				
		Total PBL		PBL	TP	RES	REC	REC-3D
1° line	**COJEC**	10/22	Cisplatin	4/11	(2-3)/7	(2-3)/7	(2-3)/7	(2-3)/7
			Vincristine	0/1	N/A	N/A	N/A	N/A
			Carboplatin	4/6	2/3	2/4	2/3	2/3
			Etoposide	1/1	N/A	N/A	N/A	N/A
			Cyclophosphamide	1/3	(1-2)/3	(1-2)/3	(1-2)/3	(1-2)/3
	**CADO**	2/5	Clophosphamide	1/3	(1-2)/3	(1-2)/3	(1-2)/3	(1-2)/3
			Adriamycin	1/1	N/A	N/A	N/A	N/A
			Vincristine	0/1	N/A	N/A	N/A	N/A
	**CARBO/VP16**	5/7	Carboplatin	4/6	2/3	2/4	2/3	2/3
			Etoposide	1/1	N/A	N/A	N/A	N/A
2° line			Topotecan	N/A	N/A	N/A	N/A	N/A
	**TOPO/EDX**		Endoxifen	N/A	N/A	N/A	N/A	N/A
3° line		1/1	Ifosfamide	N/A	N/A	N/A	N/A	N/A
	**IFO/DOXO**		Doxorubicin	1/1	N/A	N/A	N/A	N/A
4° line	**CARBO/VP16**	5/7	Carboplatin	4/6	2/3	2/4	2/3	2/3
			Etoposide	1/1	N/A	N/A	N/A	N/A
